# Catalpol Protects Against Retinal Ischemia Through Antioxidation, Anti-Ischemia, Downregulation of β-Catenin, VEGF, and Angiopoietin-2: In Vitro and In Vivo Studies †

**DOI:** 10.3390/ijms26094019

**Published:** 2025-04-24

**Authors:** Howard Wen-Haur Chao, Windsor Wen-Jin Chao, Hsiao-Ming Chao

**Affiliations:** 1 Department of Medicine, School of Medicine, Aston University, Birmingham B4 7ET, UK; 2 Department of Medical Education, Leeds University, Leeds LS2 9JT, UK; 3 Department of Science, University of British Columbia, Vancouver, BC V6T 1Z4, Canada; 4 Department of Chinese Medicine, School of Chinese Medicine, China Medical University, Taichung 404, Taiwan; 5 Institute of Pharmacology, Department of Medicine, School of Medicine, National Yang Ming Chiao Tung University, Taipei 11221, Taiwan; 6 Department of Ophthalmology, Shin Kong Hospital, Taipei 105, Taiwan

**Keywords:** catalpol, H_2_O_2_, OGD, catalpol, retinal ischemia, neuroprotection, Chinese medicine, β-catenin, VEGF, angiopoietin-2, HIF-1α, MCP-1

## Abstract

Retinal ischemic disorders present significant threats to vision, characterized by inadequate blood supply oxygen–glucose deprivation (OGD), oxidative stress, and cellular injury, often resulting in irreversible injury. Catalpol, an iridoid glycoside derived from *Rehmannia glutinosa*, has demonstrated antioxidative and neuroprotective effects. This study aimed at investigating the protective effects and mechanisms of catalpol against oxidative stress or OGD in vitro and retinal ischemia in vivo, focusing on the modulation of key biomarkers of retinal ischemia, including HIF-1α, vascular endothelial growth factor (VEGF), angiopoietin-2, MCP-1, and the Wnt/β-catenin pathway. Cellular viability was assessed using retinal ganglion cell-5 (RGC-5) cells cultured in DMEM; a 3-(4,5-Dimethylthiazol-2-yl)-2,5-diphenyltetrazolium bromide assay was performed. H_2_O_2_ (1 mM)/OGD was utilized. Vehicle or different catalpol concentrations were administered 15 min before the ischemic-like insults. The Wistar rat eyes’ intraocular pressure was increased to 120 mmHg for 60 min to induce retinal ischemia. Intravitreous injections of catalpol (0.5 or 0.25 mM), Wnt inhibitor DKK1 (1 μg/4 μL), anti-VEGF Lucentis (40 μg/4 μL), or anti-VEGF Eylea (160 μg/4 μL) were administered to the rats’ eyes 15 min before or after retinal ischemia. Electroretinogram (ERG), fluorogold retrograde labeling RGC, Western blotting, ELISA, RT-PCR, and TUNEL were utilized. In vitro, both H_2_O_2_ and OGD models significantly (*p* < 0.001/*p* < 0.001; H_2_O_2_ and OGD) induced oxidative stress/ischemic-like insults, decreasing RGC-5 cell viability (from 100% to 55.14 ± 2.19%/60.84 ± 4.57%). These injuries were insignificantly (53.85 ± 1.28% at 0.25 mM)/(63.46 ± 3.30% at 0.25 mM) and significantly (*p* = 0.003/*p* = 0.012; 64.15 ± 2.41%/77.63 ± 8.59% at 0.5 mM) altered by the pre-administration of catalpol, indicating a possible antioxidative and anti-ischemic effect of 0.5 mM catalpol. In vivo, catalpol had less effect at 0.25 mM for ERG amplitude ratio (median [Q1, Q3] 14.75% [12.64%, 20.48%]) and RGC viability (mean ± SE 63.74 ± 5.13%), whereas (*p* < 0.05 and *p* < 0.05) at 0.5 mM ERG’s ratio (35.43% [24.35%, 43.08%]) and RGC’s density (74.34 ± 5.10%) blunted the ischemia-associated significant (*p* < 0.05 and *p* < 0.01) reduction in ERG b-wave amplitude (6.89% [4.24%, 10.40%]) and RGC cell viability (45.64 ± 3.02%). Catalpol 0.5 mM also significantly protected against retinal ischemia supported by the increased amplitude ratio of ERG a-wave and oscillatory potential, along with recovering a delayed a-/b-wave response time ratio. When contrasted with DKK1 or Lucentis, catalpol exhibited similar protective effects against retinal ischemia via significantly (*p* < 0.05) blunting the ischemia-induced overexpression of β-catenin, VEGF, or angiopoietin-2. Moreover, ischemia-associated significant increases in apoptotic cells in the inner retina, inflammatory biomarker MCP-1, and ischemic indicator HIF-1α were significantly nullified by catalpol. Catalpol demonstrated antiapoptotic, anti-inflammatory, anti-ischemic (in vivo retinal ischemia or in vitro OGD), and antioxidative (in vitro) properties, counteracting retinal ischemia via suppressing upstream Wnt/β-catenin and inhibiting downstream HIF-1α, VEGF, and angiopoietin-2, together with its decreasing TUNEL apoptotic cell number and inflammatory MCP-1 concentration.

## 1. Introduction

Retinal ischemia, characterized by an insufficient blood supply to the retina and/or choroid, is a seminal factor in the pathological processes behind vision-threatening retinal disorders. As a highly metabolic tissue, the retina relies on a continuous blood supply providing nutrients and oxygen to sustain cellular function; during ischemic events, this supply is disrupted, leading to cellular stress and tissue damage. Retinal ischemia is central to conditions such as central retinal artery occlusion (CRAO), central retinal vein occlusion (CRVO), proliferative diabetic retinopathy, glaucoma, and hereditary retinal diseases (e.g., familial exudative vitreoretinopathy, Coats disease, Norrie disease) [1,2,3,4,5].

Under the conditions of ischemia—oxygen and nutrient deprivation—retinal cells enter a state of metabolic stress, activating adaptive mechanisms to conserve energy and maintain essential cellular functions. As oxygen levels further decline, cells shift towards anaerobic respiration to maintain ATP production. While this switch provides short-term metabolic support, it rapidly becomes maladaptive, as prolonged ischemia initiates a cascade of damaging processes [6]. Reduced ATP availability impairs energy-dependent ion pumps, disrupting ionic gradients that lead to cellular swelling and dysfunction. This disruption increases intracellular calcium levels, initiating excitotoxicity—a harmful process of cellular overstimulation that undermines structural integrity, while anaerobic respiration leads to lactic acid buildup, intensifying cellular stress [7].

As ischemia persists, mitochondrial function is compromised, further contributing to the buildup of reactive oxygen species (ROS). With a hampered oxygen supply, the electron transport chain (ETC) is disrupted, leading to electron leakage that generates ROS, such as superoxide anions. Additionally, upon reperfusion, the sudden influx of oxygen creates an oxidative burst, intensifying ROS production [8]. Moreover, the ischemia-induced immune response further contributes to increased ROS production. These cumulative responses damage lipids, proteins, and DNA, exacerbating retinal cell degeneration [9].

To compensate for oxygen deficiency, the retina initiates an adaptive neovascular response. Under hypoxic conditions, the cellular response is mediated by several key players, including hypoxia-inducible factor-1α (HIF-1α), placental growth factor (PLGF), retinoblastoma-binding protein 2 (RBP2), and vascular endothelial growth factor (VEGF) [5]. HIF-1α acts as a transcriptional regulator, activating genes that facilitate angiogenesis through increased VEGF expression [10]. While the angiogenic response aims to restore oxygen supply, it often becomes maladaptive in the retinal context. The newly formed vessels are structurally fragile and prone to leakage and rupture, resulting in complications, such as macular edema and hemorrhage [11]. These are hallmarks of diseases, such as retinal vein/artery occlusion, diabetic macular edema, and age-related macular degeneration.

While these pathways form the classical framework of pathological response in retinal ischemia, recent research highlights the Wnt/β-catenin signaling pathway as a pivotal player with both adaptive and maladaptive roles in ischemic retinal diseases [12]. The Wnt signaling pathway is essential for the normal development of retinal vessels; however, in ischemic conditions, it becomes dysregulated and accelerates disease progression. In the presence of hypoxia, the Wnt/β-catenin pathway is activated as a survival mechanism; β-catenin (unphosphorylated), in turn, becomes overexpressed and interacts with downstream transcriptional factors, such as HIF-1α [13], initiating a shift toward inflammatory and proangiogenic activity. This shift amplifies VEGF production, further driving neovascularization (NV) [14].

Building upon this pathway, angiopoietin-2 (Ang-2) expression has emerged as a crucial downstream mediator and a novel biomarker in ischemic retinal disease [15]. Ang-2, known for its role in destabilizing blood vessels, becomes overexpressed in response to hypoxic stress. In a balanced vascular environment, angiopoietin-1 (Ang-1) binds to the Tie-2 receptor to promote vessel stability pericyte modulation. However, as Ang-2 levels rise in ischemic conditions, it competitively inhibits Ang-1’s stabilizing effect on the Tie2 receptor, increasing vascular permeability and enhancing vessel fragility [16]. The destabilizing effect of Ang-2 exacerbates the pathological angiogenic cycle, especially in conjunction with elevated VEGF [15]. The co-expression of Ang-2 with VEGF has shown accelerated NV compared to VEGF alone, exacerbating the fragility and abnormality of the immature vessels [17]. Without intervention, this cascade results in severe complications, including ocular hemorrhage, subretinal and cystoid macular edema, and eventual retinal cell death with vision compromise.

Despite notable advancements in therapeutic treatments, such as intravitreal monoclonal antibody injections targeting vascular endothelial growth factor (anti-VEGF) and/or angiopoietin-2—including Ranibizumab (Lucentis) and Aflibercept (Eylea)—retinal ischemia remains to be a challenge in the field of ophthalmology [18]. Whilst these treatments generally help stabilize vision and reduce NV, certain severe or chronic ischemic conditions still do not adequately respond [19]. This limits the effectiveness of existing monotherapies and has driven the development of combined therapeutic approaches, such as Vabysmo, which targets both VEGF and Ang-2 to enhance treatment outcomes [20]. Human vitreous extraction studies have shown that patients with exudative age-related macular degeneration (eAMD), proliferative diabetic retinopathy, and retinal artery or vein occlusion exhibit elevated levels of Ang-2—a multifunctional cytokine involved in both angiogenesis and the regulation of inflammation [21]. Inflammatory cytokines (e.g., TNF-α, IL-1β, IL-6, IL-10, and MCP-1) might be related to retinal ischemia-related disorders, such as exudative AMD [21,22]. New scientific research in this field, including genetic and inflammatory studies in the treatment of wet macular degeneration, indicate that co-inhibiting Ang-2 and VEGF pathways leads to a significant reduction in blood vessel leakage and tumor-associated angiogenesis, offering a synergistic benefit over single-agent therapy [23]. For example, Canonica et al. (2023) reported that the dual inhibition of Ang-2 and VEGF-A notably suppressed retinal inflammation in JR5558 mice [24]. This was demonstrated by a substantial reduction in Iba1-positive microglia and macrophages clustered around CNV lesions, an outcome not achieved to the same extent with the single inhibition of either Ang-2 or VEGF-A [24]. Other genetic research on Ang-2−/− phenotype mice showed less vascular leakage in response to VEGF, while wild-type mice experienced increased leakage of VEGF and no change when treated with NaCl [25].

The recognition that retinal ischemia involves complex and multiple biological pathways highlights the need for novel treatment strategies that target multiple mechanisms. Such treatments could improve outcomes for patients who are resistant to standard monotherapies. Additionally, the requirement for frequent injections—often administered monthly or bi-monthly—presents challenges related to patient discomfort, potential side effects, and long-term adherence [26]. These limitations underscore the necessity of pursuing innovative strategies that adopt multi-targeted approaches.

Catalpol, chemically known as 1,6,7-trihydroxyxanthone glycoside, is a naturally occurring, water-soluble, iridoid glycoside sourced from *Rehmannia glutinosa*, a plant commonly referred to as the Chinese foxglove or “Di Huang” in Traditional Chinese Medicine (TCM). Valued for its anti-inflammatory and antioxidative effects, catalpol has been shown to possess anti-ischemic potential, particularly shown through its neuroprotective effects in cerebral ischemia and anti-inflammatory effects in myocardial ischemia [27,28]. In vitro, catalpol has demonstrated antioxidative effects against H_2_O_2_-induced oxidative stress in astrocytes [29] and anti-ischemic-like effects against astrocytes under OGD [30]. In vivo, catalpol has demonstrated protective effects against renal ischemia/reperfusion injury through the downregulation of inflammatory markers [31]. Catalpol has also demonstrated neuroprotective effects against strokes in rats. Moreover, the administration of catalpol mitigated the impairment of the neovascular unit post-ischemic stroke [32].

While much of the research on catalpol focuses on cardiovascular and cerebrovascular diseases, its potential applications for retinal protection are promising, given the retina’s neuronal similarities to the brain. Recent research underscores catalpol’s antioxidative and antiapoptotic properties, particularly in relevance to ischemic conditions [27,28,29,30,31,32,33,34,35,36,37,38,39,40,41]. Studies have demonstrated that catalpol can enhance the survival of neurons through neurogenesis activation and promote vascular integrity following ischemic events [30,33,35,39,41]. Consistently, catalpol has been found to increase cerebral blood flow and stimulate stroke-induced STAT3 activation, subsequently restoring STAT3 activity by facilitating its binding to VEGF [38]. Moreover, catalpol treatment was revealed to increase the expression of GAP-43 and p-S6 [37], contributing to pro-axonal regeneration and its neurorestorative effects. Further demonstrating its efficacy, catalpol has been shown to promote vascular integrity amidst neovascularization following corneal ischemia [36]. Despite these promising findings, research on catalpol’s specific effects in retinal ischemia remains limited, making our present study a novel contribution that could extend the understanding of catalpol’s potential as a retinal therapeutic agent.

As aforementioned, inflammatory markers play a critical role in ischemic-related ocular disorders, such as age-related macular degeneration (AMD), central retinal vein occlusion (CRVO), and normal-tension glaucoma. Key cytokines, including TNF-α, IL-6, and IL-1β [36], have been extensively studied in the context of ischemia reperfusion injury, with previous research demonstrating the anti-inflammatory effects of catalpol. Studies have shown that catalpol can suppress VEGF and TNF-α levels in animal models, thereby reducing pathological neovascularization and dampening inflammatory responses. While these cytokines have been the primary focus of prior research, monocyte chemoattractant protein-1 (MCP-1) is an equally critical inflammatory chemokine that warrants further investigation. Traditional studies on ischemia reperfusion injury have predominantly centered on TNF-α, IL-6, and IL-1β in the context of catalpol’s effects, as previously mentioned [36]. To build upon this existing knowledge, we have incorporated an innovative approach by exploring MCP-1’s anti-inflammatory pathway. Despite its established role in retinal inflammation, MCP-1 has received comparatively less attention in this context. It plays a pivotal role in immune cell recruitment, particularly macrophages, which drive oxidative stress, inflammation, and vascular dysfunction. Given its significance in retinal pathology, our study aimed to expand the understanding of MCP-1’s role in ischemic retinal conditions, highlighting its importance alongside TNF-α, IL-6, and IL-1β in the inflammatory cascade.

The main aim of this study is to investigate the potential neuroprotective and anti-ischemic effects of catalpol in the context of retinal ischemia, based on preliminary data previously presented as a poster and oral presentation at the 26th European Association for Vision and Eye Research (EVER) Congress [42]. This study will employ a multifaceted approach combining in vivo and in vitro studies to comprehensively assess the impact of catalpol in retinal ischemia. In vitro investigations included the use of a cell injury model, including H_2_O_2_-induced oxidative stress mode, OGD model, and MTT cell viability assay. In vivo studies included electroretinogram (ERG), the retrograde labeling of retinal ganglion cells (RGCs), TUNEL assay, Western blot, and mRNA expression studies. The present study measured the expression levels of critical retinal ischemic or related inflammatory biomarkers, such as MCP-1, β-catenin, VEGF, and Ang-2, to gain insights into the mechanisms of action of catalpol against retinal ischemia.

## 2. Results

### 2.1. Oxidative Stress

**Figure 1:**
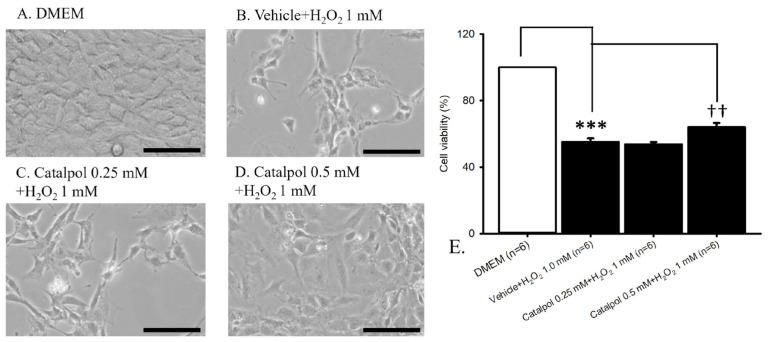
Oxidative stress model. Optical microscopy and cell viability. (**A**) Control (RGC-5 cells in DMEM) displayed numerous cells. (**B**) Incubation with 1 mM H_2_O_2_ for 24 h drastically reduced cell count. (**C**,**D**) Fifteen min pre-incubation with catalpol did not exhibit a therapeutic effect against 1 mM H_2_O_2_ ((**C**); at 0.25 mM) but resulted in a prominent protection of the cells against oxidative stress ((**D**); at 0.5 mM). (**E**) Compared to control, 24 h incubation of cells with 1 mM H_2_O_2_ significantly (*** *p* < 0.001) reduced cell viability. However, 15 min pre-incubation with 0.5 mM catalpol significantly (^††^ *p* < 0.01) ameliorated the H_2_O_2_-induced significant (*** *p* < 0.001) decrease in cell viability. Scale bar = 50 μm. Abbreviations: RGC—retinal ganglion cell; DMEM—Dulbecco’s modified Eagle medium.

Cellular viability was assessed by examining cell density under an optical microscopy (Figure 1, n = 6). In the control group (Figure 1A; RGC-5 cells cultured in DMEM), a notable population of RGC-5 cells were observed. Conversely, exposure to oxidative stress induced by the incubation of RGC-5 cells with 1 mM H_2_O_2_ (Figure 1B) for 24 h resulted in a prominent reduction in RGC-5 cell count. Notably, 15 min pre-administration of catalpol did not exhibit a therapeutic effect at 0.25 mM against 1 mM H_2_O_2_ (Figure 1C), with a more pronounced protective effect against oxidative stress observed at 0.5 mM (Figure 1D). In Figure 1E, in comparison with the RGC-5 cells cultured in DMEM (control = 100%; n = 6), exposure to 1 mM of H_2_O_2_ for 24 h led to a significant (*** *p* < 0.001) reduction in cell viability, which was not affected by pretreatment with vehicle (vehicle + H_2_O_2_ 1 mM = 55.14 ± 2.19%; n = 6). Furthermore, when compared to RGC-5 cells subjected to 24 h H_2_O_2_-induced oxidative stress, 15 min pre-administration of 0.5 mM catalpol resulted in a statistically significant (^††^ *p* = 0.003) amelioration of the H_2_O_2_-induced decrease in cell viability (catalpol 0.5 mM + H_2_O_2_ 1 mM = 64.15 ± 2.41%; n = 6). Notably, preincubation with 0.25 mM catalpol did not exhibit an improvement in cell viability (catalpol 0.25 mM + H_2_O_2_ 1 mM = 53.85 ± 1.28%; n = 6).

### 2.2. Oxygen–Glucose Deprivation (OGD)

**Figure 2:**
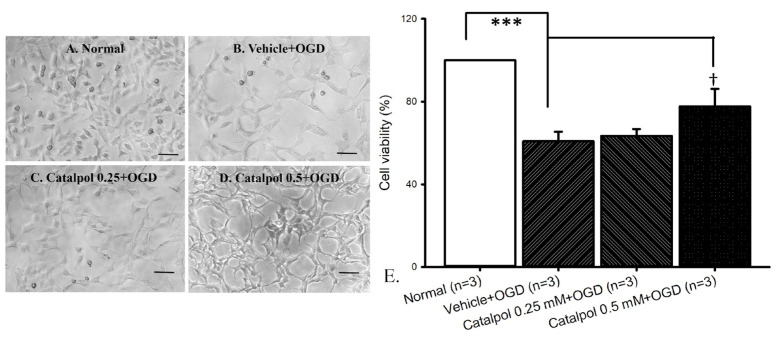
OGD model. Light microscopy was utilized to examine RGC-5 cell count. (**A**) Normal cells in DMEM. (**B**) Cells subjected to 8 h OGD and pre-administered with DMSO (vehicle + OGD) showed a considerable reduction in the cell number compared to normal. Pre-administration of catalpol demonstrated increased cell viability with greater effect at 0.5 mM (**D**) and lesser at 0.25 mM (**C**). As demonstrated in Figure (**E**), cell viability was significantly (*** *p* < 0.001; n = 3) reduced (60.84 ± 4.57%) following OGD. This reduction was significantly (^†^ *p* = 0.012) ameliorated following 15 min pre-administration of 0.5 mM catalpol (77.63 ± 8.59%). However, 15 min pre-administration of 0.25 mM catalpol (63.46 ± 3.30%) did not significantly protect cells against the OGD. The control was normalized as 100%. Scale bar = 50 μm. Abbreviations: OGD—oxygen–glucose deprivation; DMEM—Dulbecco’s modified Eagle medium.

The assessment of RGC-5 cell count was performed via light microscopy (Figure 2). The normal group consisted of cells cultured in DMEM (Figure 2A), while cells subjected to OGD with pre-administration of DMSO were defined as vehicle + OGD (Figure 2B). A noticeable reduction in cell count compared to the normal group was observed in the vehicle + OGD group. Subsequently, the impact of 15 min pre-administration of 0.25 mM (Figure 2C) and 0.5 mM (Figure 2D) catalpol on cells subjected to OGD was examined. Specifically, the protective effect of the pre-OGD administration of catalpol on the OGD was demonstrated by increased cell viabilities at 0.5 mM. The effect of 0.5 mM catalpol was found to be greater when administered 15 min before OGD (Figure 2D), with a lesser effect observed at 0.25 mM catalpol (Figure 2C).

In Figure 2E, cell viability was significantly (*** *p* < 0.001) reduced in the vehicle + OGD group (60.84 ± 4.57%; n = 3) compared to the normal group (100%; n = 3). Moreover, compared to the vehicle + OGD group, the pre-OGD administration of 0.5 mM catalpol (catalpol 0.5 mM + OGD = 77.63 ± 8.59%; n = 3) exhibited a significant (* *p* = 0.012) protective effect against OGD. However, the 15 min pre-administration of catalpol 0.25 mM (catalpol 0.25 mM + OGD = 63.46 ± 3.30%; n = 3) did not have significant (*p* = 0.55) increase in cell viabilities against OGD.

### 2.3. Electroretinogram (ERG)

**Figure 3:**
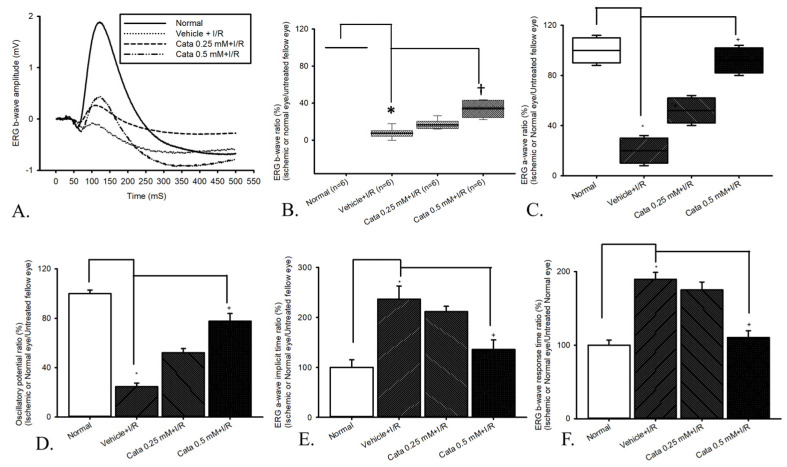
Electroretinogram (ERG): the effect of pre-ischemic catalpol intravitreous injection (IVI) on retinal ischemia plus reperfusion (I/R). (**A**) HIOP induced ischemia, and subsequent reperfusion drastically reduced ERG b-wave amplitude (vehicle + IR) compared to normal. Counteraction of this reduction was observed with pretreatment of catalpol, especially at 0.5 mM catalpol. (**B**) The vehicle + I/R group showed a significant decrease (* *p* < 0.05) in the b-wave ratio compared to the normal group. Pretreatment with catalpol was significant at 0.5 mM (^†^ *p* < 0.05) mitigated the ischemia-induced decrease in b-wave ratio. (**C**,**D**) ERG a-wave/oscillatory potential. The vehicle + I/R group showed a significant decrease (* *p* < 0.05) in the a-wave/oscillatory potential amplitude ratios compared to those of the normal group. Pretreatment with catalpol significant (^†^ *p* < 0.05) at 0.5 mM mitigated the ischemia-induced decrease in a-wave/oscillatory potential amplitude ratio. (**E**,**F**) ERG a-/b-wave response time**.** ERG a-/b-wave response time ratio was significantly delayed in the vehicle + I/R group compared to the normal group (* *p* < 0.05), indicating delayed nerve impulse conduction after I/R injury. Treatment with catalpol 0.25 mM + I/R counteracted delayed response time but did not reach statistical significance compared to the vehicle + I/R group (* *p* > 0.05). However, catalpol 0.5 mM + I/R significantly resuscitated prolonged response time compared to the vehicle + I/R group (^†^ *p* < 0.05) and approached normal levels. Abbreviations: ischemia/reperfusion, I/R.

The ERG wavefront averages were utilized to examine the retinal electrophysiological response (Figure 3). As depicted in Figure 3A, the ERG b-wave amplitude was found to be 2.04 mV in the retinas of normal rat eyes. Following retinal ischemia, a drastic reduction in b-wave amplitude was observed, unaffected by pre-ischemia treatment with vehicle (vehicle + I/R: 0.06 mV). However, pre-ischemia treatment with 0.25 or 0.5 mM catalpol (Cata 0.25 mM + I/R or Cata 0.5 mM + I/R) mitigated the ischemia-induced reduction in b-wave amplitude, raising the amplitudes to 0.32 and 0.75 mV, respectively.

As shown in Figure 3B and Table 1 (n = 6), compared to the control group, the b-wave ratio exhibited a significant (* *p* < 0.05) decrease in the vehicle + I/R group. Notably, pre-ischemic catalpol significantly (^†^ *p* < 0.05; at Cata 0.5 mM + I/R group) reduced the ischemia-induced b-wave ratio.

As demonstrated in Figure 3C, ERG a-wave amplitude ratio was significantly reduced in the vehicle + I/R group (median [Q1,Q2]: 20.0% [10.00%, 30.00%]) compared to the normal group (median [Q1, Q2]: 100% [90%, 110%]; *p* < 0.05), indicating substantial retinal dysfunction following I/R injury. Treatment with catalpol 0.25 mM + I/R (median [Q1, Q2]: 52.00% [42.00%, 62.00%]) and catalpol 0.5 mM + I/R (median [Q1, Q2]: 92.000% [82.00, 102.00%]) led to significant (*p* < 0.05; at 0.5 mM) recovery compared to the vehicle + I/R group.

As revealed in Figure 3D, ERG oscillatory potential amplitude ratio was significantly reduced in the vehicle + I/R group (24.59 ± 2.83%) compared to the normal group (100.00 ± 2.82%; *p* < 0.05). Treatment with both catalpol 0.25 mM + I/R (52.06 ± 3.37%) and catalpol 0.5 mM + I/R (77.68 ± 6.15%) led to significant (*p* < 0.05; 0.5 mM) resuscitation compared to the vehicle + I/R group.

As reported in Figure 3E,F, the ERG a-/b-wave response time ratio was significantly prolonged in the vehicle + I/R group (236.28 ± 26.46%/189.58 ± 9.24%) compared to the normal group (100.00 ± 14.95%/100.00 ± 6.81%; *p* < 0.05), indicating delayed nerve impulse conduction after I/R injury. Treatment with catalpol 0.25 mM + I/R (211.46 ± 10.71%/175.00 ± 10.76%) and catalpol 0.5 mM + I/R (135.76 ± 19.02%/110.42 ± 9.24%) led to less effect at 0.25 mM and importantly significant (*p* < 0.05; at 0.5 mM) recovery compared to the vehicle + I/R group.

### 2.4. The Effect of Catalpol on Retrograde Fluorogold Immunolabeled Retinal Ganglion Cells Density

**Figure 4:**
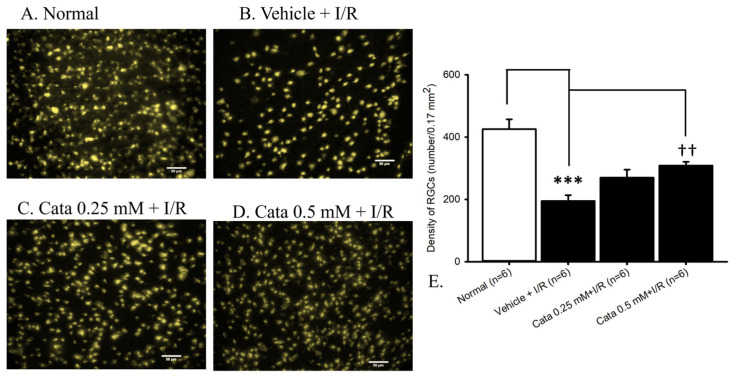
Fluorogold-labeling. The microscopic images demonstrated the density of retinal ganglion cells (RGCs). (**A**) The normal retina (normal). (**B**) Ischemic retina with 15 min pretreatment with vehicle (vehicle + I/R). (**C**,**D**) Fifteen-minute pretreatment of the ischemic retina with 0.25 mM (Cata 0.25 mM + I/R, (**C**)) and 0.5 mM (Cata 0.5 mM + I/R, (**D**)) catalpol. (**E**) Compared to normal, the density of RGCs was significantly (*** *p* < 0.001) reduced in rats subjected to retinal ischemia (vehicle + I/R). This reduction was significant (^††^ *p* < 0.01) at 0.5 mM pretreatment of catalpol. Single field area ≈ 0.17 mm^2^. Scale bar = 50 μm.

Assessing RGC density using fluorogold retrograde labeling (Figure 4; n = 6) revealed the density of the control group (normal, Figure 4A,E) to be 425.57 ± 31.47 cells/field. Compared to the control group, there was a significant (*** *p* < 0.001) reduction in RGC density (194.54 ± 18.85 cells/field) in animals subjected to retinal ischemia with pre-administered vehicle (vehicle + I/R, Figure 4B,E). Furthermore, this decrease was significantly (at 0.5 mM; ^††^ *p* < 0.01) mitigated when the animals underwent retinal ischemia with the pre-administration of catalpol (Cata 0.50 mM + I/R, Figure 4D,E: 308.50 ± 12.10 cells/field). However, a non-significant effect was observed with pretreatment with a lower concentration of 0.25 mM catalpol (Cata 0.25 mM + I/R, Figure 4C,E: 269.82 ± 25.57 cells/field).

### 2.5. Protein and mRNA Analysis

#### 2.5.1. Western Blot

**Figure 5:**
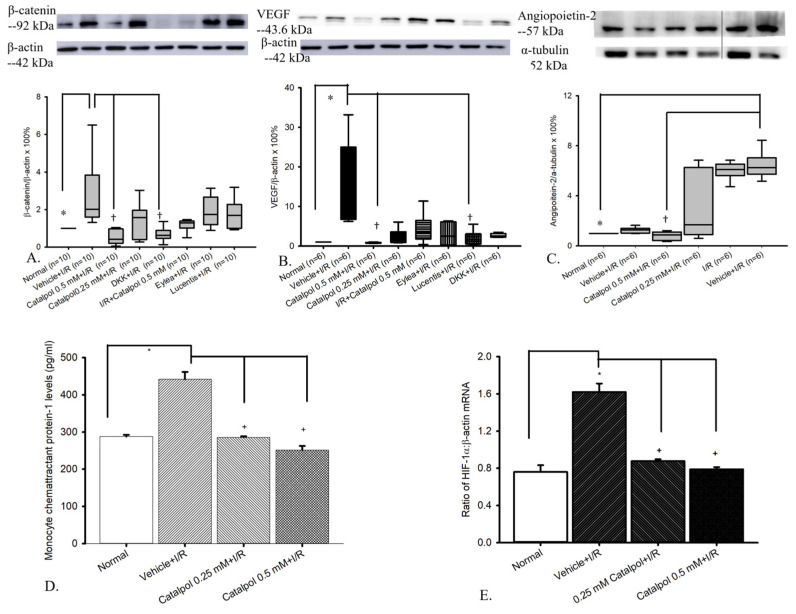
Western blot and protein expression analysis of β-catenin (**A**), VEGF (**B**), and angiopoietin-2 (**C**) relative to β-actin or α-tubulin. (**A**–**C**) Ischemia/reperfusion (I/R) insult significantly (* *p* < 0.05) increased protein expression of β-catenin, VEGF, and angiopoietin-2. This overexpression was dose-dependently mitigated with catalpol pretreatment, showing a significant reduction at 0.5 mM (^†^ *p* < 0.05 at 0.5 mM) and a lesser effect at 0.25 mM. Pre-administration with DKK1 (1 μg/4 μL, Wnt inhibitor) significantly (^†^ *p* < 0.05) attenuated β-catenin overexpression, while Lucentis (40 μg/4 μL, VEGF antibody) significantly (^†^ *p* < 0.05) inhibited the upregulation of VEGF. β-actin (42 kDa) or α-tubulin (52 kDa) were used as housekeeping proteins. Protein molecular weights: β-catenin (92 kDa), VEGF (43.6 kDa), and angiopoietin (57 kDa). A thin black line in (**C**) indicates where the gel was spliced to exclude irrelevant information—the marker and other experimental groups. (**D**) MCP-1 protein measured by ELISA. As compared to retinas subjected to pressure-induced retinal ischemia pretreated with vehicle (vehicle + I/R) to the normal retinas (control; n = 4), vehicle + I/R (n = 4) resulted in a significant (* *p* < 0.05) increase in MCP-1 inflammatory biomarker concentrations. This increase was significantly (^†^ *p* < 0.05) nullified by 15 min of pre-administration with 0.25 mM (n = 4) and 0.5 mM catalpol (n = 4). (**E**) Real-time polymerase chain reaction analysis measuring the expression of HIF-1α and β-actin. Twenty-four hours after ischemia plus reperfusion (I/R), whole retinal extracts were isolated from the normal eyes (control) or the ischemic eyes (subjected to 60-min high intraocular pressure) pre-administered before I/R with intravitreous vehicle (DMEM) or catalpol (0.25 or 0.5 mM). Ischemia reperfusion (I/R) injury resulted in a significant increase in HIF-1α protein levels (* *p* < 0.05). This elevation was significantly reduced (^†^ *p* < 0.05) after 15 min pretreatment with 0.25 and 0.5 mM catalpol. Data are presented as the mean ± SD (n = 3). Abbreviations: VEGF, vascular endothelial growth factor; HIF-1α, hypoxia-inducible factor-1α.

Regarding the ratio of β-catenin/β-actin protein, in Figure 5A and Table 2 (n = 10), the difference between normal and vehicle + I/R was significant (* *p* < 0.05). Moreover, the difference between vehicle + I/R and catalpol 0.5 mM + I/R and that between vehicle + I/R and DKK + I/R were significant (^†^ *p* < 0.05). However, the difference between vehicle + I/R and catalpol 0.25 mM + I/R, Lucentis + I/R, and Eylea + I/R, respectively, was not significant.

About the VEGF/β-actin protein, in Figure 5B and Table 3 (n = 6), the difference between normal and vehicle + I/R was significant (* *p* < 0.05). Moreover, the difference between vehicle + I/R and catalpol 0.5 mM + I/R and that between vehicle + I/R and Lucentis + I/R were significant (^†^ *p* < 0.05). However, the difference between vehicle + I/R and catalpol 0.25 mM + I/R, I/R + catalpol 0.5 mM, Eylea + I/R, and DKK + I/R, respectively, was not significant.

Regarding the angiopoietin-2/α-tubulin protein, in Figure 5C and Table 4 (n = 6), the difference between normal/vehicle + normal and vehicle + I/R or I/R was significant (* *p* < 0.05). Furthermore, pre-ischemic catalpol dose-dependently significantly (^†^ *p* < 0.05 at 0.5 mM, Cata 0.5 mM + I/R) counteracted the I/R-upregulated ang-2 levels, which was not affected by vehicle (vehicle + I/R).

**Figure 6:**
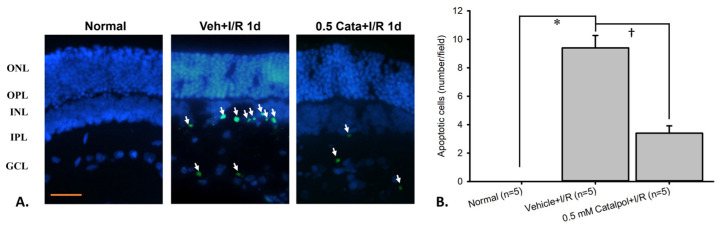
TUNEL assay. (**A**) Representative histopathological image of TUNEL-stained apoptotic cells (white arrow). (**B**) Quantification of apoptotic cells (TUNEL-positive cells) across different experimental groups. A significant increase (* *p* < 0.05) in the number of apoptotic cells was observed in the ischemic group compared to the normal group. Pre-administration of 0.5 mM catalpol significantly (^†^ *p* < 0.05) reduced the number of apoptotic cells. Scale bar 25 μm. Abbreviations: TUNEL—terminal deoxynucleotidyl transferase dUTP nick end labeling.

The impact of 0.5 mM catalpol on the presence of apoptotic cells in the inner retinal layer was assessed using the TUNEL assay. As presented in Table 1 and Figure 6 (n = 5), no TUNEL-positive cells were observed in the control group (normal; 0 cells/field). However, one day after pressure-induced retinal ischemia followed by vehicle treatment, there was a significant increase in TUNEL-positive cells (vehicle + I/R; 1.80 ± 0.37 cells/field). This marked increase in apoptosis was significantly reduced upon treatment with 0.5 mM catalpol (0.5 mM catalpol + I/R; 0.40 ± 0.25 cells/field).

#### 2.5.2. Measurement of MCP-1 Protein Using ELISA

In Figure 5D (n = 4) and Table 5, when comparing retinal cells subjected to retinal ischemia induced by HIOP with the normal retinas (287.77 ± 4.68 pg/mL; control), retinal ischemia (441.87 ± 19.58) led to significant (* *p* < 0.001) elevated MCP-1 inflammatory protein levels. This elevation was significantly (^†^ *p* < 0.001) attenuated by 15 min of pretreatment with 0.25 mM (285.58 ± 2.92) and 0.5 mM catalpol (251.54 ± 11.15).

#### 2.5.3. The Effect of Catalpol on HIF-1α mRNA in the Retina

In Figure 5E (n = 4) and Table 6, compared with the normal retina (HIF-1α = 0.76 ± 0.07)], the ratios for HIF-1α (1.62 ± 0.09) in the vehicle-pretreated ischemic retina was significantly upregulated (*p* < 0.05) after I/R. When compared with the vehicle-pretreated ischemic retina, the ischemic retina pretreated with 0.25 mM/0.5 mM catalpol (HIF-1α = 0.88 ± 0.02/0.79 ± 0.02) showed a significant (*p* < 0.05) counteraction of the I/R-induced HIF-1α upregulation.

The impact of 0.5 mM catalpol on the presence of apoptotic cells in the inner retinal layer was assessed using the TUNEL assay. As presented in Table 7 and Figure 6 (n = 5), no TUNEL-positive cells were observed in the control group (normal; 0 cells/field). However, one day after pressure-induced retinal ischemia followed by vehicle treatment, there was a significant increase in TUNEL-positive cells (vehicle + I/R; 9.40 ± 0.87 cells/field). This marked increase in apoptosis was significantly (*p* < 0.05) reduced upon treatment with 0.5 mM catalpol (0.5 mM catalpol + I/R; 3.40 ± 0.51 cells/field).

## 3. Discussion

The prevailing notion within the scientific community suggests the possibility of a shared mechanism of action across different retinal ischemic diseases. While anti-VEGF antibodies have exhibited significant efficacy in addressing ocular hemorrhage and macular edema over the past two decades, it is noteworthy that suboptimal visual outcomes persist in a subset of patients despite these treatments. Hence, the emergence of alternative drugs targeting different pathways becomes imperative.

Recent advancements in the treatment of ischemic eye diseases have led to the development of drugs that simultaneously target multiple pathways. As a result, the management of retinal ischemia increasingly involves a combination of compounds, highlighting the importance of novel therapeutic agents that extend beyond VEGF inhibition. As aforementioned, catalpol has garnered attention recently due to its antioxidative, antiapoptotic, and anti-ischemic properties. Catalpol has shown its anti-ischemic effects in protecting astrocytes, cardiac endothelium, cerebral vasculature, and corneal vasculature from ischemic damage. However, given that catalpol’s effects on retinal ischemia remain largely unexplored, our study aimed to investigate its potential impact on retinal ischemia.

### 3.1. Catalpol’s Protective Effect Against I/R, OGD, and Oxidative Stress

Retinal ischemia is fundamentally driven by oxygen and glucose deprivation, which leads to energy failure and subsequent cell death/apoptosis [43]. This is consistent with the present study’s results. The OGD model employed revealed a significant reduction in RGC-5, retinal progenitor, cellular viability, underscoring the critical impact of ischemia on retinal cells, such as RGCs. Notably, pretreatment with catalpol ameliorated these reductions, with the 0.5 mM concentrations yielding a statistically significant protective effect. Catalpol’s stabilization of cell viability under metabolically compromised conditions highlights its neuroprotective potential in ischemic/hypoxic environments.

The ischemic environment along with ischemic reperfusion drives a pro-inflammatory cascade that induces oxidative stress [44], further compounding cellular damage and accelerating retinal cell injury. Ischemia in retinal cells causes a shift to anaerobic respiration, leading to ATP depletion, disrupted ion gradients, and excitotoxicity. This results in cellular swelling and increased stress from lactic acid buildup. Furthermore, during ischemic reperfusion, ROS production is exacerbated by the sudden reintroduction of oxygen, which interacts with the damaged mitochondria and ETC to produce an oxidative burst. This surge of ROS overwhelms the cell’s natural antioxidant defenses, leading to extensive lipid peroxidation, protein oxidation, and DNA damage, further compromising the retinal cell integrity and function. This effect was reproduced in the H_2_O_2_-induced oxidative stress model, where RGC-5 cells demonstrated a marked decrease in viability. Consistent with the effects in the OGD, catalpol 0.5 mM significantly improved cell survival, demonstrating its antioxidative properties against oxidative stress.

The detrimental effects of ischemic damage are also prominent in vivo. Following I/R injury, the significant impairment of Müller/Bipolar cell’s electrophysiological function was observed, as evidenced by a significant decline or delay in ERG a-wave/b-wave/oscillatory potential amplitude (indexing photoreceptors/bipolars or Müllers/amacrines) or a-wave/b-wave response time (reflecting nerve impulse conduction velocity), alongside a significant reduction in retrograde-labeled RGC viability and increased apoptotic cells in the inner retinal layer. However, catalpol 0.5 mM was able to significantly preserve RGC density, exert antiapoptotic effects on inner retinal cells, and maintain retinal electrophysiological function. These results underscore catalpol’s ability to support the survival of various cells, while maintaining the functional integrity of retinal cells affected by ischemic injury.

From both the in vivo and in vitro results, it is evident that catalpol possesses properties that defend against I/R, OGD and oxidative stress. This is possibly explained by its antioxidative capacity, supported by You et al. (2011), who demonstrated that cultivars of *Rehmannia glutinosa* (which contain catalpol) exhibit potent antioxidative activities, with an IC_50 of 205.8 mg/g for DDPH radical scavenging activity and 38.8 mg/g for hydroxyl radical scavenging activity [45]. These findings suggest that, during IR where ROS form, catalpol could dampen the further damage that ensued by ROS during ischemic injury, through chemically reducing these ROS. This is further supported by our in vivo results where we can see a demonstrated reduced RGC cell death and decreased ERG a-wave/b-wave/oscillatory potential amplitude reduction.

The protective properties of catalpol are further supported by a comprehensive review by Zhang and colleagues (2023), which elucidated catalpol’s antioxidative and antiapoptotic properties against cardio-cerebrovascular diseases [34]. Additionally, a meta-analysis by Zheng et al. (2017) further demonstrated catalpol’s neuroprotective, antioxidative, anti-inflammatory, and antiapoptotic effects in models of cerebral ischemic stroke [35]. Moreover, Lin et al. (2024) demonstrated that catalpol was able to alleviate oxidative stress through inhibiting neuronal apoptosis following oxidative stress [33]. In addition, a large-scale analysis by Bhattamisra et al. (2019) revealed that catalpol exhibits cardioprotective, neuroprotective, anti-inflammatory, and antioxidative effects through multiple molecular mechanisms [46]. Furthermore, catalpol has been shown to provide a neuroprotective effect against brain ischemia in rats by antioxidation, antiapoptosis, and modulation of angiogenesis and neurogenesis. Altogether, these findings suggest that catalpol may be able to counteract critical mechanisms in retinal ischemic injury—energy failure and oxidative stress—by enhancing cell viability and limiting oxidative damage in a dose-dependent manner.

### 3.2. Catalpol’s Mechanism of Action: HIF-1α, β-Catenin, VEGF, Angiopoietin-2, the Wnt Signaling Pathway, and the Inflammatory Factor MCP-1 in the Ischemic Retina

A growing body of research suggests that the Wnt-signaling pathway plays a crucial role not only in the early normal development of retinal vessels but also in the progression of certain developmental retinal vascular diseases [1,2,5,15,47,48]. Furthermore, this pathway is closely associated with both upstream β-catenin and downstream angiopoietin-2/VEGF signaling. Ang-2, recognized as a pivotal proangiogenic factor [14], plays a significant role in this pathway.

Under normoxic environments, β-catenin interacts with T-cell factor-4 (TCF-4) to promote cell proliferation and maintain tissue integrity [12]. However, under the ischemic/hypoxic milieu, β-catenin undergoes a functional shift that contributes to both adaptive and pathological outcomes in retinal cells [49]. Specifically, it activates downstream hypoxia-inducible factor 1α (HIF-1α), where the overexpressed HIF-1α protein competes with TCF-4 for binding with cellular β-catenin. Upon binding to HIF-1α, β-catenin promptly shifts its role from co-activating TCF-4 to triggering HIF-1α-associated transcriptional processes [50]. This upregulation of HIF-1α-mediated transcription under ischemic situations leads to subsequent elevation in ang-2/VEGF levels. VEGF, a potent pro-angiogenic factor, plays a critical role in the formation of new vessels (neovascularization); however, under ischemic conditions, it often leads to pathological neovascularization. The newly formed vessels typically lack structural integrity, making them prone to leakage and rupture. This compromised vasculature leads to complications, such as macular edema and hemorrhage, which are hallmarks of ischemic retinal diseases, such as diabetic retinopathy and retinal vein occlusion [5,51].

Alongside VEGF, angiopoietin-2 plays a complementary but distinct role in regulating vascular homeostasis. Ang-2 functions as a context-dependent antagonist of Ang-1, which stabilizes blood vessels through its interaction with the Tie-2 receptor on pericytes [52]. Under physiological conditions, Ang-1 activates the Tie-2 receptor to maintain vascular maturation, quiescence, and barrier integrity. However, under ischemia, Ang-2 expression is significantly upregulated, disrupting the balance between Ang-1 and Ang-2 and inhibiting the stabilizing effects of Ang-1 [16]. This results in vessel destabilization, increased endothelial permeability, and enhanced sensitivity of the vasculature to VEGF signaling [53]. Consequently, the combined action of VEGF and Ang-2 further exacerbates the fragility of immature pathological vessels.

In our present study, catalpol demonstrated the ability to downregulate β-catenin, VEGF, and Ang-2 levels in retinal tissue. Specifically, catalpol at 0.5 mM significantly counteracted the upregulation of these pro-angiogenic factors. In our analysis, we compared β-catenin levels in the presence of DKK (an established inhibitor of the Wnt/β-catenin pathway), as well as VEGF levels in the presence of Lucentis (ranibizumab), a well-known anti-VEGF agent. Interestingly, catalpol exhibited a similar inhibitory effect on β-catenin levels as DKK, suggesting that catalpol may act as an effective modulator of the Wnt/β-catenin pathway. Furthermore, catalpol demonstrated comparable anti-VEGF activity to Lucentis, indicating its potential to modulate VEGF activity, in addition to its ability to inhibit Ang-2 expression. In line with these findings, Han and colleagues (2018) reported similar results in their investigation of rat corneal neovascularization, where catalpol was shown to inhibit levels of VEGF and TNF-α, reducing neovascularization and dampening inflammation. In a previous publication, Zhu et al. (2015) indicated that the suppression of TNF-α, IL-1β, IL-6, and IL-10 activities was involved in the protective effect of catalpol on I/R injury [31]. Inflammation indeed plays a vital role in ischemia-related disorders. In the present evaluation of the specific proinflammatory cytokine, monocyte chemoattractant protein-1, MCP-1, the novel results proved the consequent elevation of MCP-1 after retinal I/R, where catalpol was demonstrated to downregulate the levels of MCP-1, alleviating inflammation and protecting against retinal ischemia-related disorders, namely exudative AMD.

Moreover, catalpol demonstrated the ability to downregulate angiopoietin-2 levels in the retinal tissue. The modulation of Ang-2 by catalpol underscores its role in restoring vascular homeostasis under ischemic conditions. By attenuating Ang-2 expression, catalpol may help rebalance the Ang-1/Ang-2 axis, thereby mitigating the destabilizing effects typically observed in pathological neovascularization. This reduction in Ang-2 enhances pericyte support for the existing blood vessels and contributes to the improved structural integrity of newly formed vessels, ultimately reducing the risk of leakage (edema) and bleeding (hemorrhage). The combined mechanism of action of inhibiting upstream β-catenin, suppressing VEGF levels, and reducing Ang-2 protein levels likely contribute to its ability to protect retinal cells (i.e., retinal progenitors: RGC-5, retinal ganglion cells) and retinal electrophysiology under ischemic conditions and oxidative stress.

The present protein analyses and results strongly support the hypothesis that catalpol exerts its anti-inflammatory, antioxidative, and anti-ischemic/hypoxic properties via mechanisms of downregulating the ischemia-associated overexpression of MCP-1, β-catenin, HIF-1α, VEGF, and angiopoietin-2. These observations are corroborated by recent studies [15,31,40,41,54] and underscore the inhibition of MCP-1, anti-Wnt/β-catenin, anti-HIF-1α, anti-VEGF, and anti-angiopoietin-2 properties of catalpol.

### 3.3. In Vitro Antioxidative/Anti-Ischemic-Like and In Vivo Anti-Ischemic/Antiapoptotic Effects of Catalpol: Comparison with Other Compounds and Its Relation to MCP-1, HIF1α, β-Catenin, VEGF, and Angiopoietin-2

Given the increasing interest in TCM compounds for ocular health, it is important to understand how catalpol compares to other TCM compounds known for their neuroprotective and anti-inflammatory properties. While our research does not aim to identify a single compound capable of fully addressing retinal ischemia, it seeks to contribute to the broader understanding of how TCM compounds like catalpol can be integrated into combination therapies for retinal ischemic disorders. Combination therapies are becoming increasingly essential for managing complex and treatment-resistant conditions, such as normal tension glaucoma, exudative age-related macular degeneration (AMD), or proliferative diabetic retinopathy unresponsive to conventional therapy. For instance, in normal tension glaucoma, neuroprotective agents, like β-blockers (e.g., betaxolol) and α_2_ agonists (e.g., brimonidine), and carbonic anhydrase inhibitors (e.g., acetazolamide) may be used to mitigate optic nerve damage. Meanwhile, in exudative AMD, combining anti-VEGF therapies like ranibizumab or aflibercept with angiopoietin-2 inhibitors (Vabysmo) offers a comprehensive approach to managing vascular leakage and neovascularization. Catalpol is distinguished by its ability to target the angiopoietin-2, Wnt/β-catenin, and VEGF pathways alongside modulating the inflammatory biomarker MCP-1. Its mechanism of action alleviates ischemic injury through downregulating β-catenin and consequently downregulating HIF-1α and VEGF; moreover, it further prevents vascular leakage and retinal cell damage through downregulating angiopoietin-2 and MCP-1.

In contrast, moscatilin, a bibenzyl component of *Dendrobium nobile Lindley*, has been shown to protect RGC-5/RGCs against oxidative stress in vitro/retina ischemia in vivo by upregulating Norrin/downregulating PKM2, RBP2, HIF-1α, VEGF, and PLGF [1]. What is more, baicalein/mannitol “cytoprotects” hRPE (ARPE-19) against chronic/acute oxidative stress in vitro by downregulating VEGF/upregulating catalase [55]. Finally, S-allyl L-cysteine provided a protective effect on RGC-5 cells against oxidative stress in vitro by downregulating MCP-1, PKM2, and VEGF [56,57] and prevents RGCs from kainate-induced excitotoxicity in vivo through its antiapoptotic effect [4]. Presently, catalpol also protects the neurons in the inner retinas (e.g., RGCs) from retinal ischemia in vivo through its antiapoptotic effect [4].

Considering its clinical relevance, catalpol presents a potential therapeutic avenue for a spectrum of retinal ischemic disorders, including retinal vascular occlusion, proliferative diabetic retinopathy, neovascular AMD, and possibly, retinal developmental anomalies, like Coats’ disease. These conditions are characterized by pathological processes, such as angiogenesis, ocular hemorrhage, and macular edema, all associated with elevated β-catenin, HIF-1α, VEGF, angiopoietin-2, and MCP-1 levels. Catalpol’s unique action on multiple angiogenic pathways, including β-catenin, HIF-1α, VEGF, and angiopoietin-2, and on inflammatory biomarkers (i.e., MCP-1) suggests that these mechanisms may play key roles in treating retinal ischemia. By targeting distinct but complementary pathways involved in vascular stability and neuroprotection, catalpol could provide a novel therapeutic approach. Furthermore, combining catalpol with current treatments, such as anti-VEGF, anti-PLGF/VEGF traps, and anti-angiopoietin-2 agents, may enhance their efficacy in treating complex retinal conditions.

### 3.4. Side Effects, Limitations, and Future Directions

The safety assessment of any therapeutic compound is crucial, and extensive research has been conducted to evaluate the toxicity profile of catalpol [58,59,60]. Studies involving acute toxicity in mouse models have provided insights into its safety parameters. Dong et al. (2009) demonstrated that the short-term oral administration of catalpol did not result in any noticeable toxic symptoms [59]. Mice continued their normal feeding habits and physical activities, showing no signs of distress or physiological abnormalities [59]. For long-term evaluation, Jiang et al. (2008) investigated the effects of intravenous catalpol over a 90-day period, administering doses of 10, 20, and 40 mg/kg/day [60]. Findings revealed no significant changes in the biochemical markers or structural integrity of major organs, indicating that extended exposure to catalpol does not present substantial toxicity concerns [60]. Beyond systemic toxicity, potential effects on visual function were also explored. Electroretinography (ERG) b-wave assessments, combined with Sigma plot analysis, were used to examine any impact on retinal health. Results showed no significant differences in ERG b-wave amplitudes between catalpol-treated subjects and controls. These findings suggest that catalpol does not induce retinal toxicity and is unlikely to compromise visual function under the tested conditions. These findings suggest that catalpol is a safe compound with limited toxicity, offering additional benefits due to its multimodal mechanisms, antioxidative, and anti-inflammatory properties, making it a promising candidate for treating ischemia-related ocular disorders, such as age-related macular degeneration (AMD).

The RGC-5 cell line has been widely used to study retinal ganglion cells (RGCs), but its characterization remains controversial. Originally identified as rat-derived, it was later confirmed to originate from mice [61,62]. Additionally, inconsistencies in Thy-1 expression raise concerns about its reliability as a true RGC model [61,63]. To address these limitations, primary RGCs extracted from Wistar rat retinas were used instead, ensuring a biologically relevant model for studying key markers, such as VEGF, β-catenin, HIF-α, and MCP-1. RGC-5 cells were only used to assess viability following H_2_O_2_ exposure and oxygen–glucose deprivation (OGD), with results validated by the in vivo fluorogold staining of RGCs. Immunostaining confirmed Thy-1 localization in retinal ganglion cells, with strong reactivity in the inner plexiform layer (IPL) and ganglion cell layer (GCL) [57,64]. RT-PCR further validated Thy-1 expression in extracted retinal cells from normal controls, demonstrating that the isolated RGCs retained key molecular markers characteristic of authentic RGCs in our in vivo study.

The high intraocular pressure (HIOP) model was employed in the in vivo studies to investigate ischemia/reperfusion (I/R) injury, providing a controlled approach to acute retinal ischemia. However, it does not fully replicate chronic ischemic diseases [65,66]. While effective, findings must be cautiously interpreted regarding long-term disease progression. To ensure experimental reliability, key surgical controls were maintained, including corneal hydration, anesthesia regulation, and body temperature stabilization [66]. Compared to vascular ligation, the pressure-induced ischemia model minimized unintended damage to adjacent ocular structures, making it a more precise method for studying retinal ischemic injury. Despite its limitations, the HIOP model remains a valuable tool for investigating the complex mechanisms of retinal ischemia, offering critical insights into both cellular responses and potential therapeutic interventions [66].

In this study, catalpol was administered before I/R, representing a preventive approach. Future investigations could explore the potential of catalpol as a post-I/R treatment, expanding its application in contexts where early intervention may not be feasible. Furthermore, optimizing dosing regimens, delivery methods (e.g., oral), combination therapy (catalpol plus anti-VEGF and verteporfin photodynamic therapy), and assessing long-term outcomes could enhance our understanding of catalpol’s therapeutic potential and inform its future clinical applications.

## 4. Materials and Methods

### 4.1. In Vitro Studies

#### 4.1.1. Cell Injury Model

Six-well plates were utilized to grow RGC-5 cells, with each well including 3 × 10^5^/well [55]. The RGC-5 cell line was obtained from the American Type Culture Collection (ATCC, No. CRL-2302). The RGC-5 cell line represents a lineage of neuronal precursor cells [61]. RGC-5 cells were cultured in Dulbecco’s modified Eagle medium (DMEM) solution (1.5 mL/well) at 37 °C for 1 day to achieve the required cell number (3 × 10^5^/well) [67].

#### 4.1.2. Oxidative Stress

To simulate the oxidative stress cells undergoing retinal ischemia, an H_2_O_2_-induced oxidative stress model was employed [68,69,70]. The cells were then subjected to H_2_O_2_ (1 mM) for 24 h [67], either with or without a 15 min pretreatment of catalpol. The groups were categorized into three, the normal control group (DMEM only incubation), the experimental control group (pre-incubation of vehicle with 1 mM H_2_O_2_ to simulate oxidative stress), and the treatment groups (pre-administration: catalpol 0.25 mM + H_2_O_2_ 1 mM; catalpol 0.5 mM + H_2_O_2_ 1 mM). Cell viability was assessed by counting the cells using a hemocytometer. To evaluate the impact of catalpol on oxidative stress, the cell viability was compared among the various groups mentioned. Each experiment was carried out in triplicate.

#### 4.1.3. Oxygen–Glucose Deprivation (OGD)

To simulate retinal ischemia, an OGD model was employed to induce oxygen and glucose deprivation stress on retinal cells ischemia [71,72,73,74]. RGC-5 cells were subjected to OGD for a duration of 8 h within a controlled simulating environment. This methodology was adapted from several studies [5,75,76]. This involved culturing the cells in DMEM without glucose maintained at 37 °C and under 1% O_2_, 94% N_2_, and 5% CO_2_ (monitored via a Penguin incubator; Astec Company, Fukuoka, Japan).

The study comprised of several experimental groups, including cells treated as follows: (i) DMEM with vehicle (normal control, n = 3), (ii) 15 min of pre-OGD application with vehicle (vehicle + OGD, n = 3), (iii) 15 min of pre-OGD application with 0.25 mM catalpol (Cata 0.25 mM + OGD, n = 3), and (iv) 15 min of pre-OGD application with 0.5 mM catalpol (Cata 0.5 mM + OGD, n = 3). Following 8 h of OGD, the cultured cells were transferred to a new DMEM for another day. Cell viability assessment was conducted utilizing MTT assays, and the cell number was counted by a hemocytometer or cellular morphology examined under optical microscopy.

#### 4.1.4. 3-(4,5-Dimethylthiazol-2-yl)-2,5-Diphenyltetrazolium Bromide (MTT) Assay

Following incubation with either vehicle (control) or catalpol, the treated cells were washed. MTT (0.5 mg/mL; 3-(4,5-Dimethylthiazol-2-yl)-2,5-diphenyltetrazolium bromide; Sigma-Aldrich, St. Louis, MA, USA) was then introduced to the 24-well plates containing the original 400 μL cells (4 × 10^4^/well) and left to incubate for 3 h at 37 °C, as instructed by Mosmann (1983) [77]. The reduced MTT (blue formazan) was then dissolved in 200 μL dimethyl sulfoxide. After agitation, the optical density (OD) of the solubilized formazan was measured using an ELISA reader (Synergy H1 Multi-Mode Reader BioTek Instruments, Santa Clara, CA, USA) at 562 nm. Cell viability was determined by comparing the OD values to those of the control (100%; cells cultured in DMEM).

### 4.2. In Vivo Studies

#### 4.2.1. Animals

The Institutional Animal Care and Use Committee at Cheng Hsin General Hospital (CHGH; Taipei, Taiwan; Approval No: CHIACUC 106-04) consented to all the animal experiments that followed the Association for Research in Vision and Ophthalmology Statement for the Use of Animals in Ophthalmology and Vision Research. Wistar rats (200–250 g; equally sex distributed; National Laboratory Animal Center, Taipei, Taiwan) were housed with 40–60% humidity and at 19–23 °C. These rats were subjected to a 12 h light/dark cycle with 12–15 air exchanges per hour. They had access to food and water ad libitum [1,2,55,56]. Animals were excluded from the study if they exhibited significant anatomical abnormalities, were pregnant, displayed abnormal physiology, or did not survive the procedure. The researchers were blinded to group allocation throughout the experiment and data analysis to minimize bias.

#### 4.2.2. Drug Administration

Drug or vehicle administration was conducted via intravitreal injection across various groups [2]. Animals were randomly assigned to each group. These groups included pre-ischemic administration with different concentrations of catalpol (Merck, Darmstadt, Germany; 50839; 96% purity HPLC), namely, 0.25 mM catalpol (Cata 0.25 mM + I/R) and 0.5 mM catalpol (Cata 0.5 mM + I/R). Rats in the vehicle group, subjected to ischemia, received an identical volume of vehicle (vehicle + I/R) as the treatment groups. Additionally, certain groups of ischemic retinas received Lucentis (40 μg/4 μL; anti-VEGF antibody; ranibizumab; Novartis, Taipei, Taiwan), Eylea (160 μg/4 μL; anti-VEGF/PLGF antibody; aflibercept; Bayer, Taipei, Taiwan), and Wnt3a inhibitor Dkk1 (1 μg/4 μL; Abcam Inc., Cambridge, UK, ab281791; 95% purity HPLC) 15 min prior to retinal ischemia [5]. The rats were then sacrificed with an overdose (at least 140 mg/kg) intraperitoneal injection of pentobarbitone sodium; this was also carried out for the normal retina.

#### 4.2.3. Anesthesia and Euthanasia

Animal anesthesia was induced through intraperitoneal injection of 100 mg/kg of ketamine (Pfizer, New York, NY, USA) with 5 mg/kg xylazine (Sigma-Aldrich, Saint Louis, MO, USA) to minimize animal suffering during retinal ischemia induction, ERG recordings, and retrograde labeling of RGCs. The animals were sacrificed one day after retinal ischemia/reperfusion [1]. Euthanasia was thoughtfully (Scientific Procedures Acts 1986) performed by the intraperitoneal injection of at least 140 mg/kg pentobarbitone sodium (SCI Pharmtech, Taoyuan, Taiwan).

#### 4.2.4. Ischemia Induction

Following the administration of anesthesia, the Wistar rats were positioned within a stereotaxic frame. The anterior chamber of the eye under study was cannulated with a 30-gauge needle (Becton, Dickinson and Company, Franklin Lakes, NJ, USA) connected to an elevated balanced salt solution reservoir (BSS^®^ PLUS; Alcon, ZG, Zug, Switzerland). The salt reservoir was elevated at 163 cm to induce an intraocular pressure of 120 mmHg [66,78,79,80]. The presence of a pale fundus confirmed the induction of an ischemic insult [1,2,3,4,5]. The animals were placed on 37 °C heating pads during the ischemia induction. To prevent microleakage and ensure the lubrication of the eyes, hypromellose was applied to both eyes, with reapplication as required approximately every 30 min. No procedures were performed on the control eyes. IOP measurements were performed at baseline and 1 day post-I/R using a tonopen (Oculab, Inc., Glendale, CA, USA). Animals exhibiting leaks or significant drops in IOP were excluded from this study; however, no such cases were encountered in this investigation.

#### 4.2.5. ERG Recording

ERG recordings were performed to evaluate the retinal electrophysiological response, specifically the function of the inner retinal layers, including Müller and bipolar cells. Prior to the ERG recordings [1,2,4,5], the Wistar rats were subjected to >8 h of dark adaptation. Pupil dilation was achieved with topical 1% tropicamide and 2.5% phenylephrine (Alcon, ZG, Switzerland), and topical 0.5% proparacaine (Alcon, ZG, Switzerland) was applied to numb the ocular surfaces. Immediately before the ERG, anesthesia was administered, as mentioned in Section 4.2.3. of in vivo studies. ERG data were collected on all animals before and after ischemic insult (with or without intravitreal injections of vehicle or catalpol). The animal was then placed in a stereotaxic frame, and the body temperature was maintained at 37 °C with a heated water jacket. A platinum wire loop was placed on the corneal surface to act as the recording electrode. A ground electrode was connected to the scruff of the back, and a reference electrode to the tongue of the animal. A strobe positioned 2 cm distal to the rat’s eye provided a stimulus at a frequency of 0.5 Hz and a brightness of 2.1 candelas/m^2^. Fifteen consecutive responses were collected at 2 s intervals and at 10 kHz; the responses were amplified using a P511/regulated power supply 107/stimulator PS22 (Grass-Telefactor; AstroNova, Brossard, QC, Canada). To facilitate the comparison, the oscillatory potential amplitude ratio together with a-wave and b-wave amplitude ratio/response time ratio of the treated ischemic eye was compared and standardized to that of the untreated contralateral normal eye.

#### 4.2.6. Retrograde Labeling of RGCs

To dye the RGC cells, fluorogold retrograde labeling was employed. The rats were secured onto a stereotactic frame after anesthesia, as described in Section 4.2.3 of in vivo studies, then two small holes were made into the skull 1.5 mm lateral to the midline and 6 mm posterior to the bregma. This was facilitated by a prior 2 cm incision to the scalp. Fluorogold (5%; 2 μL; Sigma-Aldrich) was injected through the burr holes via a micropipette at depths of 3.8, 4.0, and 4.2 mm below the skull [81].

The micropipette was kept in each position for 3 min to prevent dye reflux. After injection, antibiotic ointment was applied to the wound, and the skin was sutured. Retinal ischemia induction was carried out on the studied eye 72 h following retrograde labeling. The animals were sacrificed, as illustrated in Section 4.2.3, one day after retinal ischemia/reperfusion.

The eyes were subsequently enucleated utilizing a stitch at the 12 o’clock position to ensure proper anatomical orientation. The eyes were then fixed in 4% paraformaldehyde for 60 min. Following the dissection of the anterior segment and the removal of the vitreous body, the whole retina was meticulously isolated and washed with 0.1 M PBS. Following fixation, the whole retina was mounted on a slide, separated evenly into four quadrants, and flat-mounted onto slides to prepare the retinal whole mounts. The slides were allowed to air-dry before being treated with DPx agent (Fluka Chemical, Ronkonkoma, NY, USA). Each quadrant of the retina was subdivided into three regions, central, middle, and peripheral, located at distances of 1, 2, and 3 mm from the optic disc, respectively. Within each region, six microscopic areas of 0.430 × 0.285 mm^2^ each along the central line were analyzed. In total, 72 areas spanning the entire retina were analyzed. The average RGC density was calculated and defined as the ratio of the total number of RGCs to the entire retinal surface area. Each microscopic field was saved to the computer, and cell numbers were manually counted on the screen by experienced technicians using a double-blind method and a hemocytometer [82]. The manual counts were also cross-referenced with automated cell counting performed using ImageJ software (version 1.54b) [83].

#### 4.2.7. Protein and MRNA Analysis

##### Western Blotting Analysis

The retinas were harvested immediately after sacrifice. The retinal tissues were then isolated and emulsified in the ultrasound in a lysis buffer containing a mammalian protein extraction reagent (HyCell, Taipei, Taiwan). Identical amounts of denatured protein (40 μg/30 μL/well) were separated using sodium dodecyl sulphate polyacramide gel electrophoresis (SDS-PAGE; Bio-Rad, Hercules, CA, USA) on a 10% separating gel and a 5% stacking gel containing 0.1% sodium dodecyl sulfate [55]. The separated proteins were transferred to a polyvinylidene difluoride membrane (Millipore, Burlington, MA, USA). The membranes underwent a blocking process for 1 h using 5% fat-free skimmed milk (Fonterra, Taoyuan, Taiwan) in PBS. Following this, the membranes underwent overnight soaking at 4 °C with primary antibodies: rabbit anti-β-catenin monoclonal antibody (ab32572; 1:5000; Abcam Inc., Cambridge, UK), mouse anti-β-actin monoclonal antibody (MW: 42 kDa; ab6276; 1:10,000; Abcam Inc., Cambridge, UK), mouse monoclonal α-tubulin (MW: 52 kDa; ab7291; 1:10,000; Abcam Inc., Cambridge, UK), rabbit anti-angiopoietin 2 polyclonal antibody (MW: 57 kDa; PA5-27297; 1:500; Thermo Fisher Scientific, Waltham, MA, USA), and mouse anti-VEGF monoclonal antibody (MW: 43.6 kDa; NB100-648; 1:1000; Novus Biologicals, Centennial, CO, USA). Subsequently, the blots were soaked for 60 min at 37 °C with secondary antibodies: horseradish peroxidase-conjugated goat anti-rabbit, or anti-mouse IgG (1:2000; Santa Cruz Biotech, Santa Cruz, CA, USA) at 37 °C for 60 min. A total of 4% fat-free skimmed milk was used to dilute the antibodies. UVP ChemStudio PLUS (Analytik Jena GmbH, Jena, Germany) was utilized to acquire and evaluate the immunoblot images. ImageJ version 1.53 t was utilized to conduct densitometric analysis [2]. The levels of β-catenin, VEGF, and Ang-2 in each sample were measured relative to that of the control group (normalized to 100%).

##### ELISA for MCP-1

MCP-1 levels in retinal cell supernatants were quantified using enzyme-linked immunosorbent assay (ELISA) kits from R&D Systems (Minneapolis, MN, USA; catalog no. DCP00). Following the manufacturer’s protocol, all assays were performed in triplicate to ensure accuracy. The average optical density of each sample was measured using a Synergy H1 Multi-Mode reader from BioTek Instruments, and MCP-1 concentrations were expressed in picograms per milliliter (pg/mL) based on the calculated standard curve.

##### HIF-1α mRNA Levels via RT-PCR

The mRNA concentrations of retinal HIF-1α were measured by rtPCR [84]. One day after a retinal ischemic insult and pre-ischemia administration of the defined chemicals or the normal (control) group, the animals were killed, and the retinal tissues were retrieved. This was followed by sonication in the presence of Tri Reagent (Sigma, St. Louis, MO, USA). Total retinal RNA was isolated, and first-strand complementary DNA (cDNA) synthesis was performed on 2 μg deoxyribonuclease (DNase; 0.05 U/μL; Promega))-treated RNA using High-Capacity RNA-to-cDNA Master Mix (Applied Biosystems, Waltham, MA, USA). The first strand cDNA then underwent real-time PCR using Fast SYBRR Green Master Mix (Bio-protech, Gangwon-do, Republic of Korea). The PCR was initiated by incubation at 95 °C for 20 s; then, 40 cycles of 95 °C for 3 s and 60 °C for 30 s were performed. Cycling was carried out on a StepOnePlus Real-Time PCR System (Applied Biosystems, Waltham, MA, USA). Relative quantification (a comparative method) was performed using the housekeeping gene β-actin as the internal standard. This procedure allowed for the measurement of the normalized level of the target mRNA and considers the differences in the quantity of total mRNA applied to each reaction (ΔCt = Ct target − Ct β-actin; cycle threshold, Ct). The relative HIF-1α expression changes caused by ischemia or the normal (control) group were calculated as fold changes relative to the contralateral untreated control normal retina using the calibration equation (Ct = ΔCt induced − ΔCt normal). The relative quantification of gene expression was calculated according to the method 2^−ΔΔCt^, as described in the manufacturer’s instructions, and was carried out by the accompanying software (RQ, ver. 1.3). The PCR reagents, software, and machine were purchased from AB Applied Biosystems. The data obtained for each treatment were pooled, and a total percentage change relative to the control was calculated. The PCR oligonucleotide primers were obtained from MISSION BIOTECH, Cambridge, MA, USA, as follows: β-actin: forward, 5′-GAACCGCTCATTGCCGATAGTG-3′; reverse, 5′-TTGTC CCTGTATGCCTCTGGTCG-3′; HIF-1α: forward, 5′-ACAGCTCCCCAGCAT TTCAC-3′; reverse, 5′-GGACAAACTCCCTCACCAAAAA-3′.

#### 4.2.8. TUNEL [Terminal Deoxynucleotidyl Transferase (TdT)-Mediated dUTP Nick End Labeling] Assay

One day after retinal ischemia, the eyes were enucleated for TUNEL staining (In situ Cell Death Detection Kit, Fluorescein; Roche; Mannheim, Germany) to detect cell apoptosis [81]. The tissue was then fixed with 10% formaldehyde for 24 h. The retinal sections were treated with proteinase K (25 g/mL) and incubated in H_2_O_2_/methanol for 5 min at room temperature to inactivate endogenous peroxidases. Corresponding negative (without dUTP) and positive control (DNase-I-treated) sections were also measured. After rinsing with Tris-buffered saline, the samples were incubated in a TdT enzyme/labeling reaction mix at 37 °C for 90 min. This reaction was based on the binding of digoxigenin–dUTP to the 30’-OH end of DNA by TdT, followed by incubation with an anti-digoxigenin antibody conjugated with peroxidase. Following the termination of the labeling reaction in stop buffer, the sections were processed in a standard streptavidin–horseradish peroxidase (HRP) reaction with 3,3′ diaminobenzidine as the chromogenic peroxidase substrate and also counterstained with methyl green. Assessment of the sections was undertaken at a magnification of 40 (Zeiss, Oberkochen, Germany). Six microscopic fields from each eye made up of three adjacent areas on both sides of the optic nerve head from each eye (ONH; 1 mm away from ONH) were used to count the TUNEL-positive cells in the inner retinal layer. The average number of TUNEL positive cells per field was used for the analysis [81].

#### 4.2.9. Statistical Analysis

The graphs in Figure 1, Figure 2, Figure 3, Figure 4, Figure 5 and Figure 6 were plotted using Sigma Plot 12.5 (Jandel Scientific, Corte Madera, CA, USA), and the program SPSS (Version 20.00) was utilized for the present statistical analysis. Analysis of variance (ANOVA) was performed to compare three or more independent groups. If the normality test indicated a normal distribution of the data, one-way ANOVA was used. The outcomes were presented as mean ± standard error. If not, non-parametric ANOVA was applied, in which the results were expressed as median and quartiles. Statistical significance was established at a probability level below 0.05 (*p* < 0.05).

## 5. Conclusions

In conclusion, our study highlights the emerging therapeutic potential of catalpol, particularly its role in targeting the Wnt/β-catenin pathway to address retinal ischemia. Our findings indicate that ischemic insults significantly impact retinal apoptosis, viability, and function; however, the pre-administration of catalpol effectively attenuates these detrimental effects. Specifically, catalpol demonstrates antioxidative, anti-inflammatory, antiapoptotic, and anti-ischemic properties, preserving cell viability and maintaining retinal function in both in vitro and in vivo models. Furthermore, our results suggest that catalpol exerts its protective effects, at least in part, via downregulating the upstream Wnt/β-catenin pathway and subsequently reducing downstream HIF-1α/angiopoietin-2/VEGF/MCP-1 expressions. This multifaceted approach highlights the potential of targeting defined ischemic cascade alongside complementary therapies, such as catalpol, in managing retinal ischemic disorders.

## Figures and Tables

**Table 1:** (**A**) Electroretinogram b-wave. The effect of catalpol on retinal ischemia/reperfusion ^1^. (**B**) Electroretinogram a-wave ratio. The effect of catalpol on retinal ischemia/reperfusion ^2^. (**C**) Electroretinogram oscillatory potential ratio. The effect of catalpol on retinal ischemia/reperfusion ^3^. (**D**) Electroretinogram a-wave response time ratio. The effect of catalpol on retinal ischemia/reperfusion ^4^. (**E**) Electroretinogram b-wave response time ratio. The effect of catalpol on retinal ischemia/reperfusion ^5^.

(A)				
Gr	Normal	Vehicle + I/R	Cata 0.25 mM + I/R	Cata 0.5 mM + I/R
M	100%	6.89% *	14.75%	35.43% ^†^
Q1	100%	4.24%	12.64%	24.35%
Q3	100%	10.40%	20.48%	43.08%
**(B)**				
**Gr**	**Normal**	**Vehicle + I/R**	**Catalpol 0.25 mM + I/R**	**Catalpol 0.5 mM + I/R**
M	100%	20.00% *	52.00%	92.00% ^†^
Q1	90%	10.00%	42.00%	82.00%
Q3	110%	30.00%	62.00%	102.00%
**(C)**				
	**Normal**	**Vehicle + I/R**	**Catalpol 0.25 mM + I/R**	**Catalpol 0.5 mM + I/R**
	100.00 ± 2.82%	24.59 ± 2.83% *	52.06 ± 3.37% ^†^	77.68 ± 6.15% ^†^
**(D)**				
	**Normal**	**Vehicle + I/R**	**Catalpol 0.25 mM + I/R**	**Catalpol 0.5 mM + I/R**
	100.00 ± 14.95%	236.28 ± 26.46% *	211.46 ± 10.71%	135.76 ± 19.02% ^†^
**(E)**				
	**Normal**	**Vehicle + I/R**	**Catalpol 0.25 mM + I/R**	**Catalpol 0.5 mM + I/R**
	100.00 ± 6.81%	189.58 ± 9.24% *	175.00 ± 10.76%	110.42 ± 9.24% ^†^

(A) ^1^ In comparison with the control group, the b-wave ratio exhibited a significant (* *p* < 0.05) decrease in the vehicle + I/R group at I/R day 1. Of note, pre-ischemic catalpol mitigated the ischemia-induced b-wave ratio reduction, with a modest effect observed at 0.25 mM and a significant effect at 0.5 mM (^†^ *p* < 0.05; Cata 0.5 mM + I/R group). Abbreviations: M, median; Q1, first quartile; Q3, third quartile; I/R, ischemia/reperfusion. (B) ^2^ In comparison with the control group, the a-wave ratio exhibited a significant (* *p* < 0.05) decrease in the vehicle + I/R group at I/R day 1. Of note, pre-ischemic catalpol alleviated the ischemia-induced a-wave ratio reduction, with a lesser effect observed at 0.25 mM and a significant improvement at 0.5 mM (^†^ *p* < 0.05; catalpol 0.5 mM + I/R group). Abbreviations: M, median; Q1, first quartile; Q3, third quartile; I/R, ischemia/reperfusion. (C) ^3^ In comparison with the control group, the oscillatory potential ratio exhibited a significant (* *p* < 0.05) decrease in the vehicle + I/R group at I/R day 1. Of note, pre-ischemic catalpol alleviated the ischemia-induced reduction in oscillatory potential ratio, with a modest effect observed at 0.25 mM and a significant effect at 0.5 mM (^†^ *p* < 0.05; catalpol 0.5 mM + I/R group). Abbreviations: I/R, ischemia/reperfusion. The results are presented as mean ± SE. (D) ^4^ In comparison with the control group, the a-wave response time ratio was significantly (* *p* < 0.05) prolonged in the vehicle + I/R group at I/R day 1. Of note, pre-ischemic catalpol attenuated the ischemia-induced elongation of the a-wave response time ratio, with a smaller effect at 0.25 mM and a significant effect at 0.5 mM (^†^ *p* < 0.05; catalpol 0.5 mM + I/R group). Abbreviations: I/R, ischemia/reperfusion. The results are presented as mean ± SE. (E) ^5^ In comparison with the control group, the b-wave response time ratio was significantly (* *p* < 0.05) delayed in the vehicle + I/R group at I/R day 1. Notably, pre-ischemic catalpol mitigated the ischemia-induced delay in b-wave response time ratio, with a less pronounced effect at 0.25 mM and a significant effect at 0.5 mM (^†^ *p* < 0.05; catalpol 0.5 mM + I/R group). Abbreviations: I/R, ischemia/reperfusion. The results are presented as mean ± SE.

**Table 2:** Western blotting. The ratios of the protein levels of β-catenin divided by that of β-actin ^§^.

Gr	1	2	3	4	5	6	7	8
M	100%	200.3% *	41.3% ^†^	156.9%	62.5% ^†^	129.0%	173.4%	168.1%
Q1	100%	159.9%	20.7%	40.4%	46.9%	101.0%	119.6%	100.2%
Q3	100%	382.3%	97.1%	159.4%	88.8%	138.3%	265.6%	227.1%

^§^ There were eight groups (n = 6 each): (1) normal, (2) vehicle + OGD, (3) catalpol 0.5 mM + OGD, (4) catalpol 0.25 mM + OGD, (5) DKK + OGD, (6) OGD + catalpol 0.5 mM, (7) Eylea + OGD, and (8) Lucentis + OGD. The differences in median values between the normal and vehicle + OGD groups, as well as between the vehicle + OGD and catalpol 0.5 mM + OGD groups, were greater than would be expected by chance and were statistically significant (* *p* < 0.05 and ^†^ *p* < 0.05, respectively). A significant difference (^†^ *p* < 0.05) was also observed between the vehicle + OGD and DKK + OGD groups. Abbreviations: M, median; Q1, first quartile; Q3, third quartile; OGD, oxygen–glucose deprivation.

**Table 3:** Western blotting. The ratios of the protein levels of VEGF divided by that of β-actin.

Gr	1	2	3	4	5	6	7	8
M	100%	1866.3% *	87.6% ^†^	139.2%	333.7%	251.9%	126.6% ^†^	258.1%
Q1	100%	674.1%	73.3%	92.4%	176.7%	23.2%	51.1%	225.6%
Q3	100%	2499.3%	93.1%	359.1%	645.1%	612.7%	307.2%	316.6%

There were 8 groups (n = 6), i.e., 1 (normal), 2 (vehicle + OGD), 3 (catalpol 0.5 mM + OGD), 4 (catalpol 0.25 mM + OGD), 5 (OGD + catalpol 0.5 mM), 6 (Eylea + OGD), 7 (Lucentis + OGD), and 8 (DKK + OGD). The differences in the median values between the normal and vehicle + OGD groups and between the vehicle + OGD and catalpol 0.5 mM + OGD groups were greater than would be expected by chance; there was a statistically significant difference (* *p* < 0.05 and ^†^ *p* < 0.05). A significant difference (^†^ *p* < 0.05) also existed between vehicle + OGD and Lucentis + OGD. Abbreviations: M, median; Q1, first quartile; Q3, third quartile; OGD, oxygen–glucose deprivation.

**Table 4:** Western blotting. The ratios of the protein levels of angiopoietin-2 divided by that of α-tubulin.

Gr	1	2	3	4	5	6
M	100%	127.5%	88.4% ^†^	169.2%	609.8%	626.1% *
Q1	100%	101.8%	42.5%	89.8%	561.9%	572.9%
Q3	100%	140.0%	110.9%	627.6%	653.8%	703.6%

There were 8 groups (n = 6), i.e., 1 (normal), 2 (vehicle + normal), 3 (catalpol 0.5 mM + OGD), 4 (catalpol 0.25 mM + OGD), 5 (OGD), and 6 (vehicle + OGD). The differences in the median values between the normal and vehicle + OGD groups and between the vehicle + OGD and catalpol 0.5 mM + OGD groups were greater than would be expected by chance; there was a statistically significant difference (* *p* < 0.05 and ^†^ *p* < 0.05). Abbreviations: M, median; Q1, first quartile; Q3, third quartile; OGD, oxygen–glucose deprivation.

**Table 5:** Analysis of the expression of MCP-1 protein levels (pg/mL) by ELISA.

Group	Normal	Vehicle + I/R	0.25 mM Catalpol + I/R	0.5 mM Catalpol + I/R
MCP-1	287.77 ± 4.68	441.87 ± 19.58 *	285.58 ± 2.92 ^†^	251.54 ± 11.15 ^†^

Analysis of the expression of MCP-1 protein levels by ELISA. Cells were extracted and isolated from the normal retinas (control) or the ischemic retinas pre-administered with vehicle or 0.25/0.5 mM of catalpol. Retinal ischemia resulted in a significant (* *p* < 0.05) upregulation of MCP-1 protein levels. This significant elevation was significantly (^†^ *p* < 0.05) attenuated by 15 min of pretreatment with 0.25 or 0.50 mM catalpol. Results are displayed as the mean ± SE (*n* = 4).

**Table 6:** The ratios of mRNA expression levels of HIF-1α to that of β-actin.

Group	Normal	Vehicle + I/R	0.25 mM Catalpol + I/R	0.5 mM Catalpol + I/R
HIF-1α/β-actin	0.76 ± 0.07	1.62 ± 0.09 *	0.88 ± 0.02 ^†^	0.79 ± 0.02 ^†^

Total mRNA was extracted and isolated from the normal retinas (control) or the ischemic retinas pre-administered with vehicle, or 0.25/0.5 mM of catalpol. Quantitative analysis is presented at 1 day after I/R. * and ^†^ denote significant differences between the normal and vehicle + I/R groups, as well as between the vehicle + I/R and 0.25/0.5 mM catalpol + I/R groups, respectively. The findings are presented as the mean ± SE (n = 4).2.6. Terminal Deoxynucleotidyl Transferase-Mediated dUTP Nick End Labeling (TUNEL).

**Table 7:** Terminal deoxynucleotidyl transferase-mediated dUTP nick end labeling assay.

Group	Control	Vehicle + I/R	0.5 mM Catalpol + I/R
Apoptotic cell No. (n = 5)	0	9.40 ± 0.87 *	3.40 ± 0.51 ^†^

No TUNEL-positive cells were observed in the normal (control) group. In contrast, the number of apoptotic cells was significantly increased at 1 day after ischemia/reperfusion (I/R) with vehicle pretreatment (vehicle + I/R) compared to the control group. Pretreatment with 0.5 mM catalpol (0.5 mM catalpol + I/R) significantly reduced the number of apoptotic cells in the inner retinal layer compared to the vehicle + I/R group. Quantitative analysis is presented at 1 day after I/R. * and ^†^ denote significant differences between the normal and vehicle + I/R groups, as well as between the vehicle + I/R and 0.5 mM catalpol + I/R groups, respectively. Abbreviation: TUNEL, terminal deoxynucleotidyl transferase-mediated dUTP nick end labeling.

## Data Availability

The data that support the findings of this study are available from the corresponding author upon reasonable request.
